# Fuel mixing enhancement of transverse coaxial air and fuel jet by upstream shock wave on in scramjet engines: numerical study

**DOI:** 10.1038/s41598-023-45810-z

**Published:** 2023-10-28

**Authors:** Seyyed Amirreza Abdollahi, Moharram Jafari, Saman Aminian, M. Fattahi, P. D. Uyen

**Affiliations:** 1 Faculty of Mechanical Engineering, University of Tabriz, Tabriz, Iran; 2 Department of Civil Engineering, College of Engineering, Cihan University-Erbil, Erbil, Iraq; 3 Institute of Research and Development, Duy Tan University, Da Nang, Vietnam; 4 School of Engineering and Technology, Duy Tan University, Da Nang, Vietnam

**Keywords:** Aerospace engineering, Mechanical engineering

## Abstract

In this study, computational fluid dynamics (CFD) is used to disclose the impacts of upstream shock waves on fuel mixing of cross coaxial air and fuel jet at a scramjet engine. This study has tried to investigate the impact of three different lobe injectors (2-lobe, 3-lobe, and 4-lobe nozzle) on the fuel penetrations along the scramjet combustor. The supersonic air stream is M = 4 while cross hydrogen and air jet are released in sonic velocity. This study uses CFD simulations to analyze the effects of upstream shock waves on fuel mixing in the transverse coaxial jet and assess their potential for improving combustion efficiency. The results demonstrate that the usage of upstream shock waves significantly increases shock interactions and augments the vortex region downstream of the jet. Our results show that the impacts of shock waves on the penetration of fuel jet released from the coaxial lobe nozzle are substantial.

## Introduction

A shock generator is a device that is used to enhance the mixing of a transverse jet in a supersonic or hypersonic flow. It is typically a wedge-shaped or ramp-shaped device that is placed in the flow upstream of the transverse jet^1–4^. When the supersonic or hypersonic airflow passes over the shock generator, it creates a series of oblique shock waves^5,6^. These shock waves compress and heat the flow, increasing the density and temperature of the flow^7,8^. The compression and heating of the flow cause the mixing rate between the fuel and air in the mixing layer to increase [7, 9, and 10]. The shock generator can also create a recirculation zone behind it, which can further enhance mixing by promoting the interaction between the fuel and air. The recirculation zone creates a region of low velocity behind the shock generator, which allows the fuel more time to mix with the air before it is burned^11,12^.

The effectiveness of a shock generator depends on several factors, including the geometry of the device, its placement in the flow, the flow conditions, and the material properties of the device^13–15^. The shock generator must be carefully designed and optimized to ensure that it enhances mixing without causing unwanted flow disturbances or pressure losses^16,17^.

While a shock generator can be an effective tool for enhancing mixing in a transverse jet, there are potential drawbacks that should be considered^18,19^. The presence of a shock generator in the flow can increase the overall drag of the system, which can reduce the efficiency of the engine^20,21^. This is particularly true if the shock generator is not optimized for minimum drag. The compression and heating of the flow by the shock generator can lead to increased heat transfer to the surface of the vehicle or engine^22,23^. This can cause thermal stresses and can reduce the lifespan of the components. The presence of a shock generator can lead to pressure losses in the flow, which can reduce the overall performance of the engine^24,25^. The design and implementation of a shock generator can be complex and require significant engineering effort^26,27^. This can increase the cost and development time of the engine system. The effectiveness of a shock generator can be limited if the flow conditions are not well-matched to the design of the shock generator. In some cases, the use of a shock generator may not provide significant benefits over simpler mixing enhancement techniques^28,29^. Overall, the use of a shock generator can be an effective tool for enhancing mixing in a transverse jet, but it is important to carefully consider the potential drawbacks and trade-offs in the design and implementation of the system^30,31^.

A shock generator is a device used to enhance the mixing of a transverse jet in a supersonic or hypersonic flow^32,33^. In a transverse jet, a stream of fuel is injected perpendicular to the main flow stream, which creates a turbulent mixing layer that enhances the mixing of the fuel with the air^34,35^. However, in high-speed flows, the mixing process can be incomplete due to the limited time available for mixing before the fuel is burned. This is where a shock generator can help. A shock generator is typically a wedge-shaped or ramp-shaped device that is placed in the flow upstream of the transverse jet. As the supersonic or hypersonic airflow passes over the shock generator, it creates a series of oblique shock waves, which compress and heat the flow^36,37^. The compression and heating of the flow cause the density and temperature of the flow to increase, which in turn increases the mixing rate between the fuel and air in the mixing layer. The shock generator can also create a recirculation zone behind it, which can further enhance mixing by promoting the interaction between the fuel and air. The recirculation zone creates a region of low velocity behind the shock generator, which allows the fuel more time to mix with the air before it is burned^38–40^.

Overall, the use of a shock generator can significantly enhance the mixing of a transverse jet in a high-speed flow, leading to improved combustion efficiency and engine performance. However, the design of the shock generator is critical, and it must be carefully optimized to ensure that it enhances mixing without causing unwanted flow disturbances or pressure losses^41,42^.

Although numerous investigations have been done for the improvement of the fuel mixing in the combustion chamber, the role of the shock generator in the mixing of the coaxial lobe injector has not been analyzed in a supersonic combustion chamber. In this study, the usage of the coaxial single jet under the impacts of the shock generator is investigated. Three types of lobe injectors are chosen to determine the impacts of nozzle types on the performance of the air and fuel jets within the combustion chamber. Different aspects of the flow are analyzed to attain the main advantages of this injection system inside the combustor.

## Governing equations and simulation methodology

The main governing equations for the modeling of high-speed flow with transverse fuel jets are RANS equations^43–46^. The energy equation is also coupled to the RANS equations since several shock waves are produced inside the combustion chamber. Besides, the ideal gas assumption of the estimation of the compressible flow is still a reasonable choice. The species transport equation is also solved because of the secondary gas of hydrogen as fuel. The turbulence characteristics of the flow are modeled by the SST turbulence model. Meanwhile, the density-based algorithm is chosen because of the high-pressure gradient in our model^47–50^. More facts regarding the governing equations have been presented in fuel details in published articles^51–53^. Theoretical methods have extensively used for optimization and improvement of the mechanical systems^54–57^.

**Figure 1:**
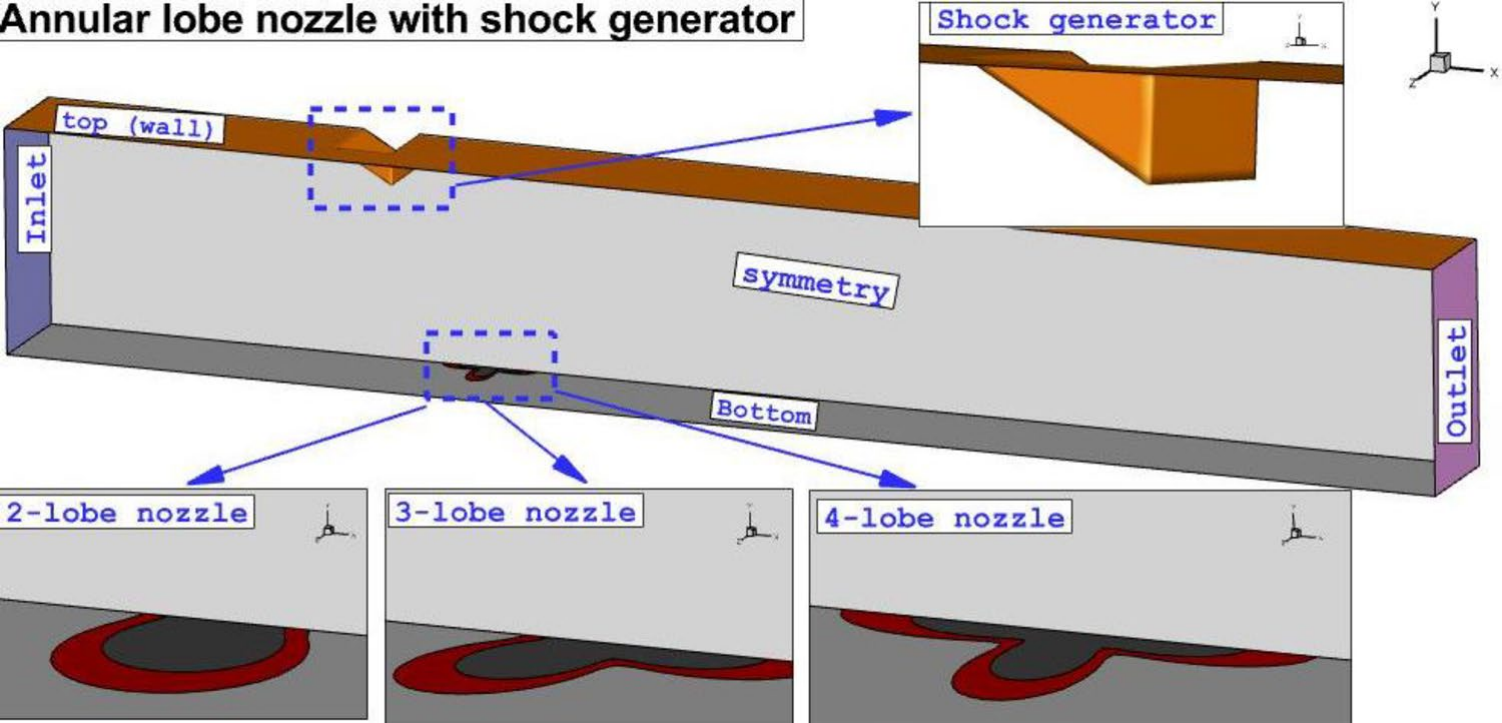
Proposed injection system.

As demonstrated in Fig. 1, three-lobe injectors are investigated in the present study and the surface areas of these models are constant to ensure the mass flow rate of these models is the same. Besides, the air jet injected from the inner nozzle to improves the fuel mixing in the combustor. The shock generator is positioned at the top of the injector and the angle of the wedge for the shock generation is 30°. To reduce the computation of the domain, half of the model is chosen for the simulation and the symmetry boundary is applied on the mid-plane as illustrated in Fig. 1. Air stream with Mach = 4, Tinf = 1000 K, and P = 1 atm is entered from the inlet and the hydrogen gas is released with Mach = 1 and total pressure of fuel jet is 10% of the total pressure of incoming airflow. The air jet is applied with the same condition as the fuel jet from the inner nozzle.

**Figure 2:**
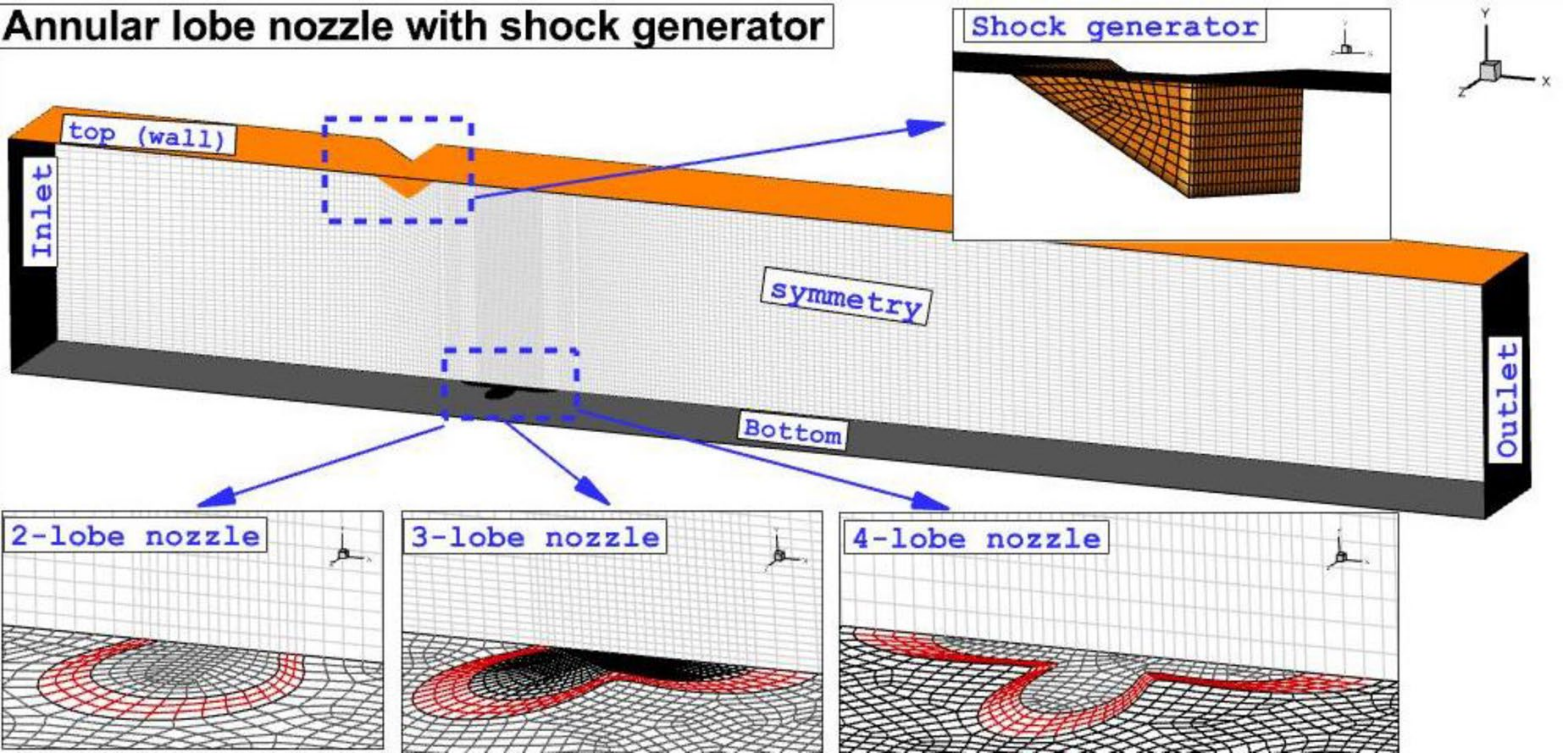
Grid production and boundary condition.

The grid is required for the simulation of the flow in the finite volume approach. As depicted in Fig. 2, the applied grid is hexagonal and structured. The homogeny grid is applied in our domain while its concentrations vary based on the importance of the region in which grid resolution near the injector and the wedge shock generator is higher because of contacts of jet flow with cross air flow. Grid analysis is done to obtain a minimum grid number for the reduction of the computational time in the simulations. In Table 1, the fuel concentrations on the plane for four grids are compared.

**Table 1:** Grid study.

	Cells	Grid cells. along X, Y and Z direction	Hydrogen fraction at 35 mm downstream
Coarse	943,100	176 × 110 × 50	0.262
Medium	1,610,100	196 × 140 × 60	0.281
Fine	2,223,100	218 × 170 × 70	0.287
Very fine	3,800,100	242 × 200 × 80	0.288

## Results and discussion

The validation is also done in this study by comparing the fuel mixing height on the symmetry plane behind the injector. Table 2 the variation of the penetration height behind the circular nozzle with diameter of 2 mm at supersonic free stream (M = 4). The results confirm the precision of the applied method for the simulation of the selected problem.

**Table 2:** Validation of penetration height (mm).

Distance from injector (mm)	Our data	Numerical data of Pudsey^43^	Errors (%)
5	5.7	6	5
10	7.05	7.1	1
20	8	7.8	2.5
30	8.4	8.2	2.5

**Figure 3:**
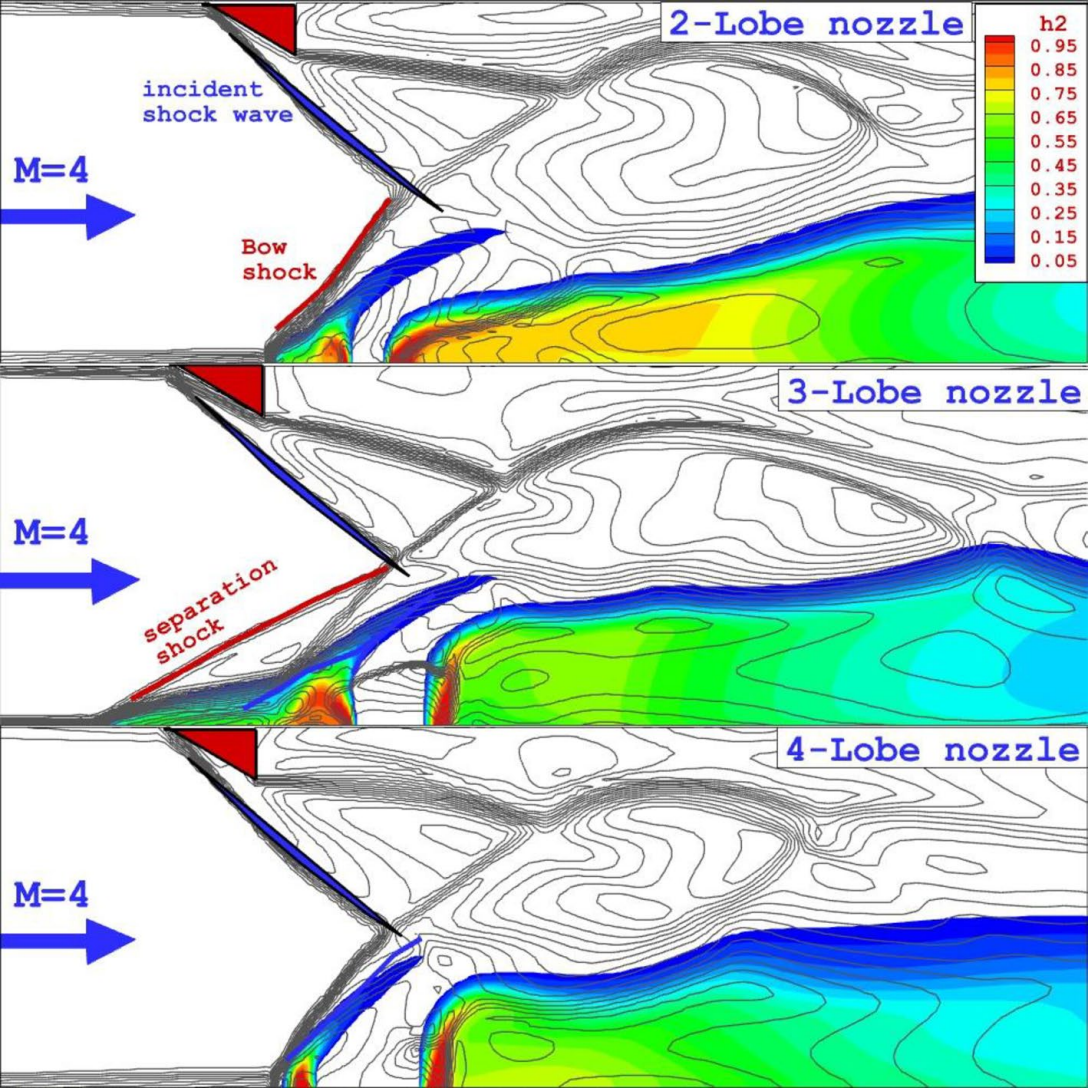
The Mach feature and Mixing zone on the symmetry plane.

The influence of the shock waves produced by the wedge upstream of the injector on the mixing zone and shock interactions is demonstrated in Fig. 3. The injection of the inner air jet on the fuel mixing and deflection of the fuel jet is also observed in this figure. Bow shock and separation shock is the key element of this configuration. The barrel shock near the nozzle is also deflected by the application of the shock generator. As the 2-lobe nozzle is replaced by the 4-lobe nozzle, the inner air jet area in the mid-section is expanded and the angle of the bow shock is also increased. The formation of separation shock for the 3-lobe injector is the main distinctive feature related to this nozzle type. In the following section, the source of this unique structure will be explained.

**Figure 4:**
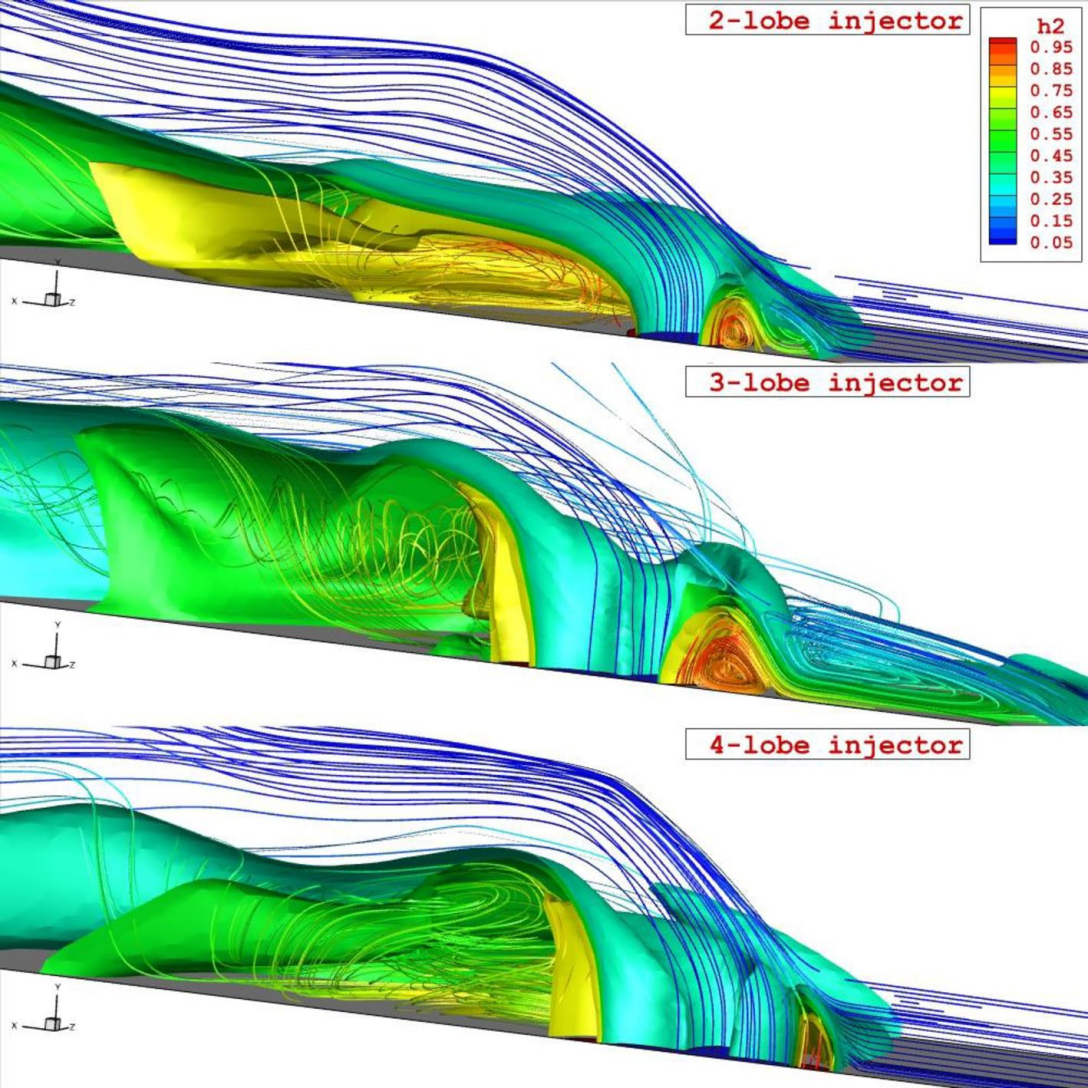
3-D structure of annular injector system under impact of shock generator.

Figure 4 demonstrates the 3-D flow feature related to the proposed configurations under the influence of the shock wave produced by the wedge upstream. Flow analysis and mass concentration layer indicate that the injection of the air from the inner nozzle improves the normal penetration of the fuel since the jet layer is distributed in a ring shape. As the number of the lobe in the injector has increased, the distribution of the fuel jet is done in the wider area and the curvature feature of the nozzle helps the formation of the vortex inside the domain.

**Figure 5:**
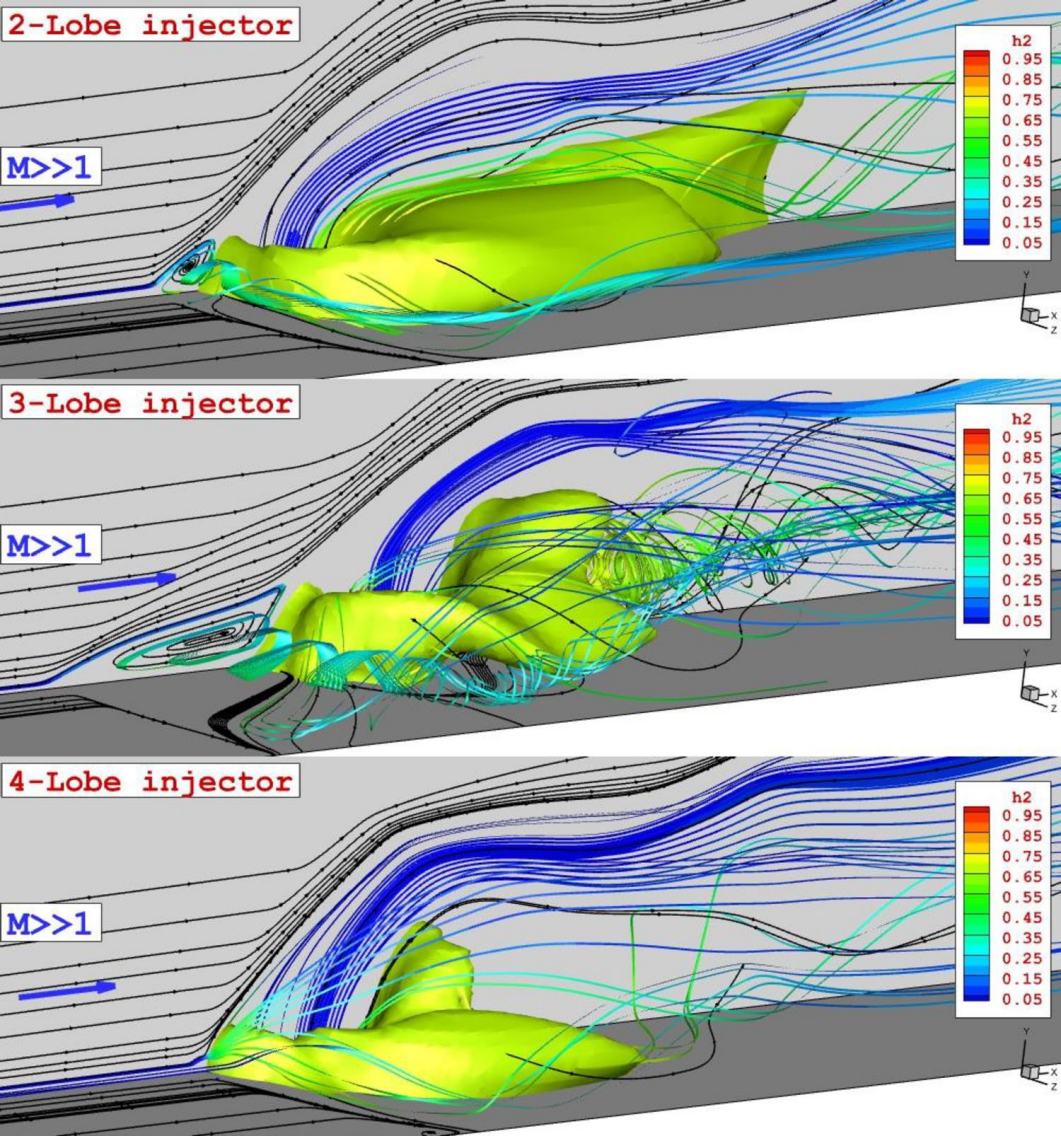
Vortex structure and shock interaction of proposed configurations.

The deflection of the air stream and formation of a vortex nearby the injector for the analysis of the fuel penetrations inside the domain is also done in this research. Figure 5 illustrates the stream of incoming supersonic flow faced with coaxial air and fuel jet. The upstream circulation is noticed upstream of coaxial fuel and air nozzle which is the first effect of the jet interactions. The largest upstream circulation is noticed in the 3-lobe nozzle. Since trails of the horseshoe vortex originally start from this vortex, the expansion of this region is effective in the mixing and distribution of the fuel. The usage of the shock generator along the inner air jet reduces the supersonic air velocity behind the fuel jet and this increases the fuel diffusion in the combustion chamber. Besides, the fuel jet layer is deformed under the impacts of the horseshoe vortex.

**Figure 6:**
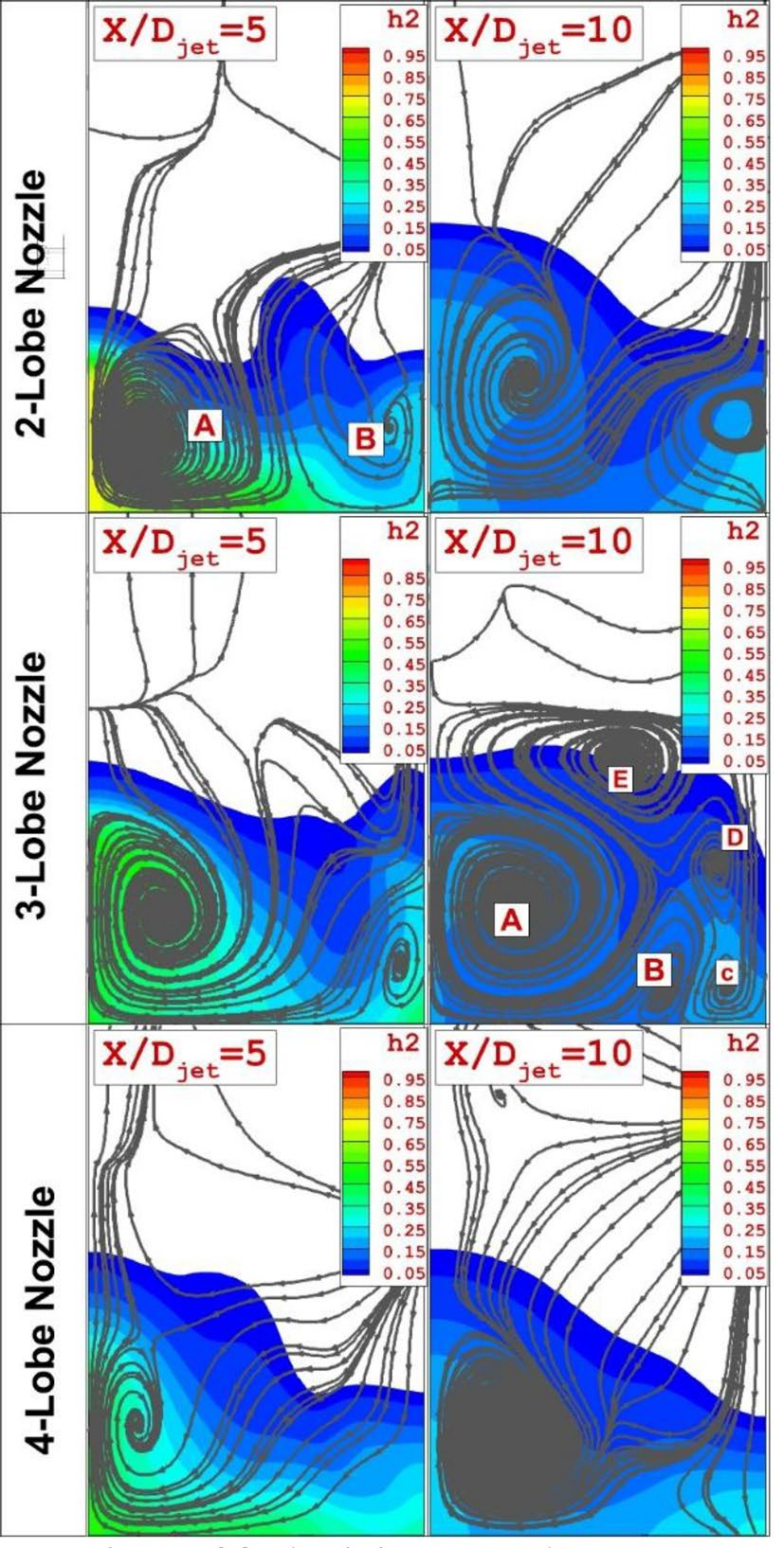
Comparison of fuel mixing zone downstream of the jets.

Figure 6 compares the mixing zone and vortex downstream of the nozzle jet for the investigated nozzle types when an upstream shock generator is applied. The main circulation is produced by the rotating vortex induced by the fuel jet and it is dominant by the reason of the interaction of the fuel jet boundary with the supersonic free stream. The secondary vortex is generated by the upstream circulation. The strength of the secondary vortex links with the power of this circulation and the archived results indicates that the power of this vortex has a significant influence on the expansion of the mixing zone. In the case of the 3-lobe nozzle, the secondary vortex may split into several vortices far downstream (x/D = 10) of the injector.

**Figure 7:**
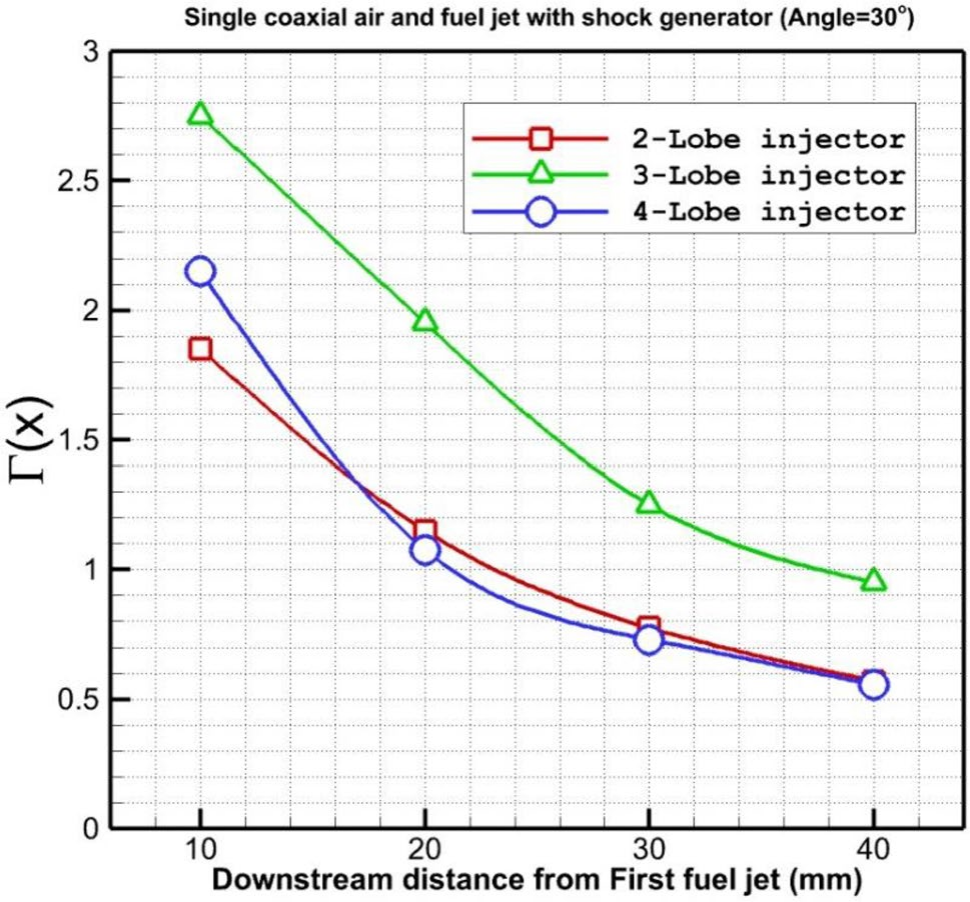
Comparison of circulation strength behind the injectors.

**Figure 8:**
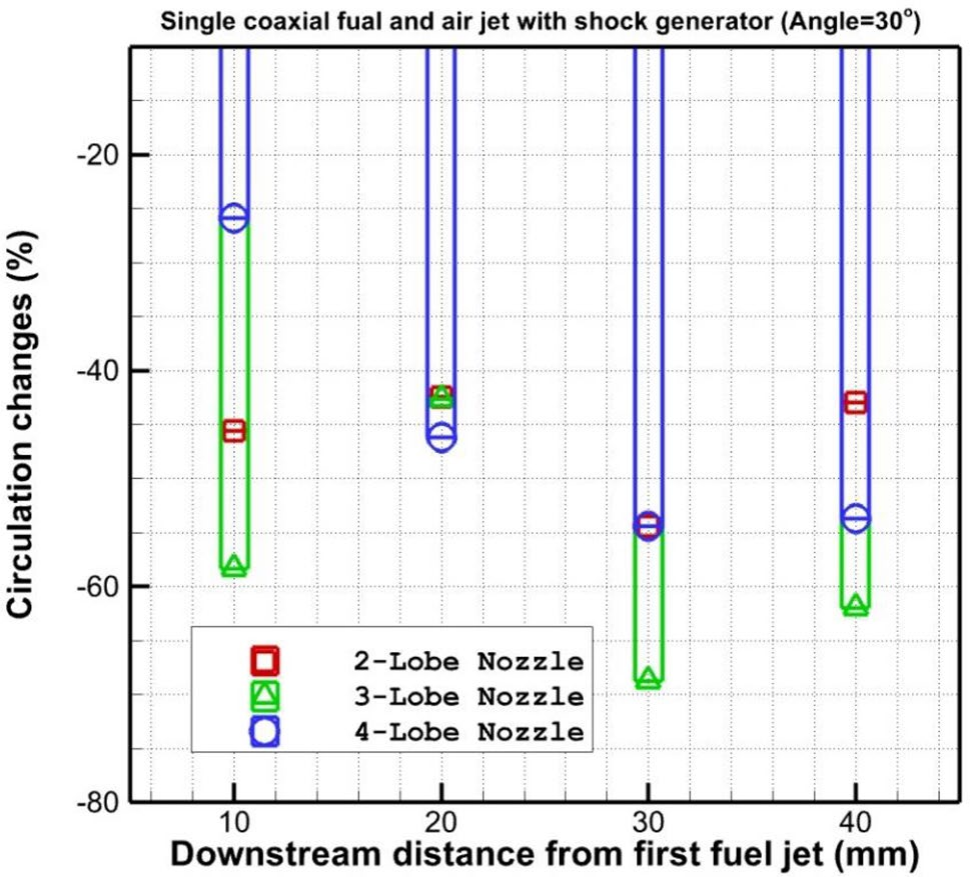
Comparison of circulation strength changes behind the injectors.

Figure 7 shows the changes in the circulation strength behind the coaxial fuel and air jet for introduced lobe-type nozzles when a shock generator is applied. The strength of circulation for the selected injectors indicates that the strength of the 3-lobe injector is about two times of other models. In Fig. 8, the influence of the upstream shock wave produced by the shock generator on the power of the circulation is demonstrated. The results show that the shock generator declines the strength of the circulation and the maximum effect is observed in the 3-lobe injector. The main reason for this reduction is associated with the reduction of the strength of the supersonic air stream with the fuel jet.

**Figure 9:**
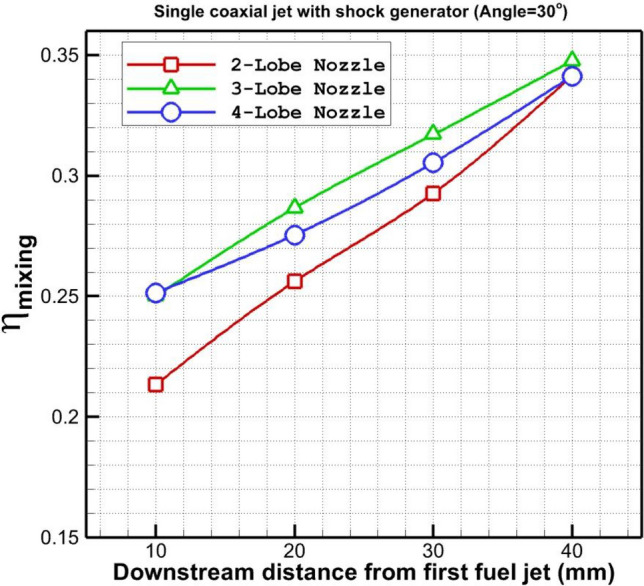
Comparison of fuel mixing performance behind the injectors.

**Figure 10:**
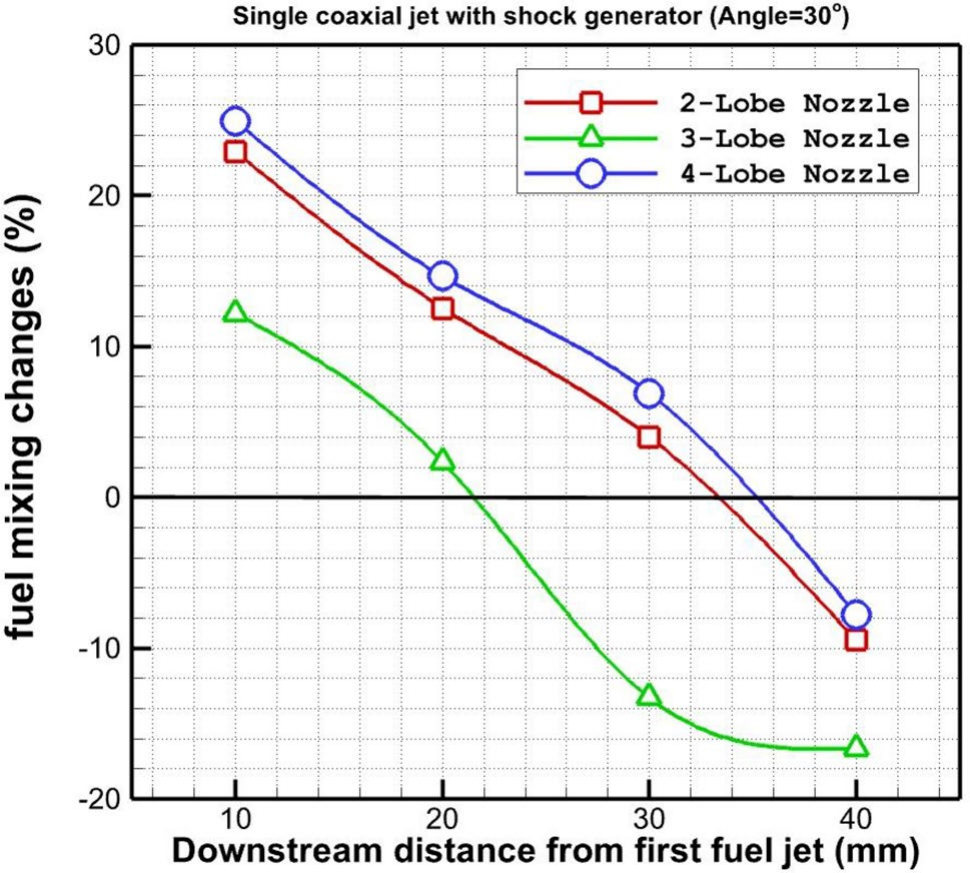
Comparison of fuel mixing performance changes behind the injectors.

In Fig. 9, the mixing efficiency of the fuel jet which is released via an annular injector with an inner air jet from different lobe injectors is plotted. The mixing performance of these nozzle configurations is almost identical far downstream since the usage of the shock generator decreases the effects of nozzle types. The main difference between these injectors is noticed close to the injector. As shown in the figure, the mixing performance of 4-lobe and 3-lobe injectors is higher than the 2-lobe one. Figure 10 disclosed the impacts of the shock generator on the variation of the fuel mixture for the introduced lobe nozzles. The changes in mixings efficiency show that the usage of the shock generator improves the fuel mixing near the nozzle while it has negative impacts far downstream. Since the vortex structure is extended by reducing the velocity of the supersonic airflow, fuel mixing and diffusion enhances in the vicinity of the injector. However, this impacts declines due to the reduction of the shock impacts downstream.

## Conclusion

The present investigation has tried to demonstrate the effects of the shock generator on the fuel mixing of the single annular jet with the inner air jet at the combustion chamber of the scramjet engine. The computational technique of CFD is applied for the analysis of the flow and jet interaction with air flow to find the mechanism of the fuel mixing inside the combustion chamber. The 3-D feature of the jet fuel is investigated to reveal the formation of different vortices behind the lobe-type injectors. In addition, the power of the circulation behind the injectors has been analyzed and it is perceived that the existence of the upstream shock generator decreases the strength of the circulation nearby the fuel jet nozzle. The comparison of the fuel mixing with/without the shock generator visibly shows that the presence of the shock generator has increased the fuel mixing near the fuel injector while the fuel mixing decreases in the far downstream. The analysis of the flow illustrates that the shock waves allow the secondary vortex to expand and consequently, the diffusion of the fuel into the main stream is increased. In afar downstream, the strength of the secondary vortex is weakening and the fuel mixing for this reason is limited.

## Data Availability

All data generated or analysed during this study are included in this published article.
